# Minimally Invasive Radiologic Uretero-calycostomy; a salvage procedure for late transplant rejection ureter necrosis

**DOI:** 10.1590/S1677-5538.IBJU.2016.0386

**Published:** 2017

**Authors:** Erich K. Lang

**Affiliations:** 1 Department of Radiology, Tulane School Medicine, New Orleans, LA, USA

Uretero-calycostomy is a time-honored procedure that has primarily been advocated for the management of failed pyeloplasties associated with long segment upper ureter strictures (1-3). We have expanded this concept to serve as salvage procedure in patients with necrosis of the transplant ureter as consequence of late rejection.

After satisfactory function of a cadaver transplant kidney for 4 years, this 36-year old female presented in the emergency room with evidence of rapidly progressing renal failure. Examination revealed 3+ edema of lower extremities, orthopnea, chest X ray bilateral pleural effusions, and laboratory findings: creatinine 14, BUN 52, K 4,8, urine output 240mL/qd, ultrasonogram showing hydronephrosis of the right transplant kidney.

**Figure 1:**
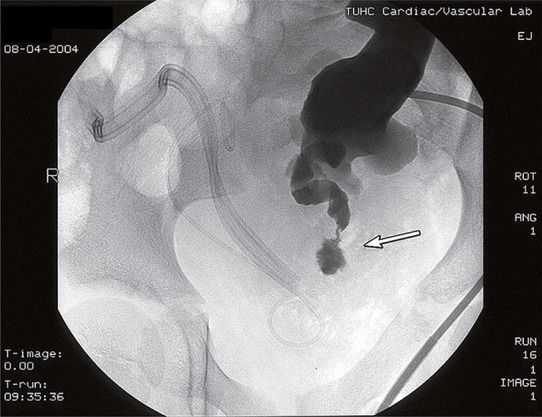
PA view: A percutaneous antegrade nephro-ureterogram demonstrates stenosis and ulcerations (arrow) in the dilated segment of the right transplant ureter. A double ”J” stent is seen in the left transplant ureter, likewise an attempt to foster healing of this ulcerated ureter. Avascular necrosis of ureter

A percutaneous antegrade nephro-ureterogram demonstrated hydronephrosis of the right transplant kidney and strictures as well as ulcerated segments of the right transplant ureter (Figure-1). Necrosis of the transplant ureter as sequel of late rejection was suggested.

**Figure 2:**
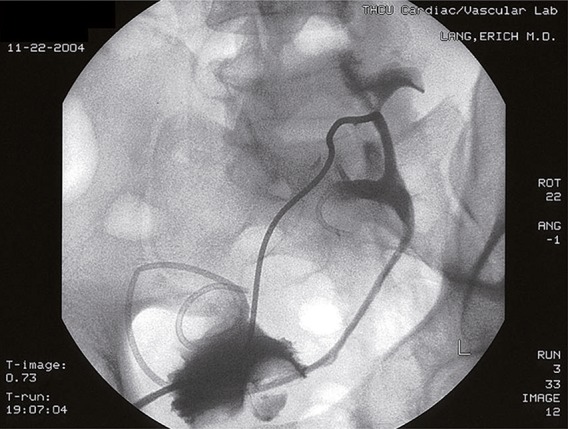
PA view: A stent has been seated via antegrade percutaneous approach from the transplant kidney pelvis into the bladder. The nephrostomy (arrow) is maintained to ensure ready access. Double J stent seated and percutaneous nephrostomy seated

Conservative management by percutaneous antegrade stent placement, anti-microbial therapy and corticosteroids failed to improve condition of the ureter (Figure-2). The uretero-neocystomy dehisced and a urinoma formed at the uretero-neocystostomy site.

Small bowel interposition to re-establish drainage or replacement of the transplant kidney by a new transplant were considered as remedial actions (4-6).

Considering the dismal condition of the transplant ureter, we decided to modify the uretero-calycostomy procedure, by creating a fistula to the native right ureter, which was available since the native right kidney had been retained for erythropoetin production. Most importantly, the native ureter was not at risk of rejection.

**Figure 3:**
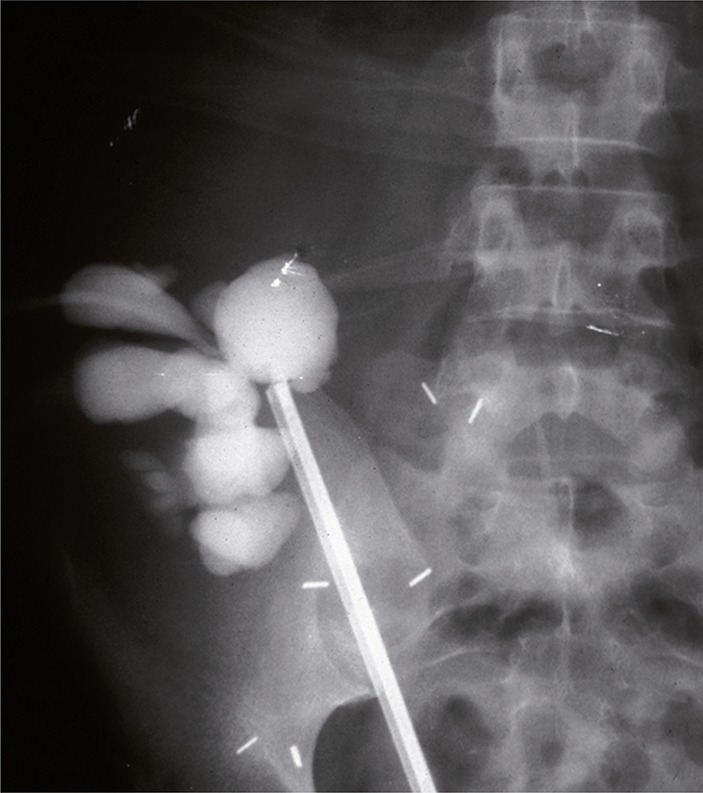
AP view: A rigid ureteroscope has been advanced in the native ureter under fluoroscopic guidance close to the dilated hydrocalyx of the right transplant kidney. The pelvis is accessed by needle-puncture through the ureteroscope and a guide wire introduced.

**Figure 4:**
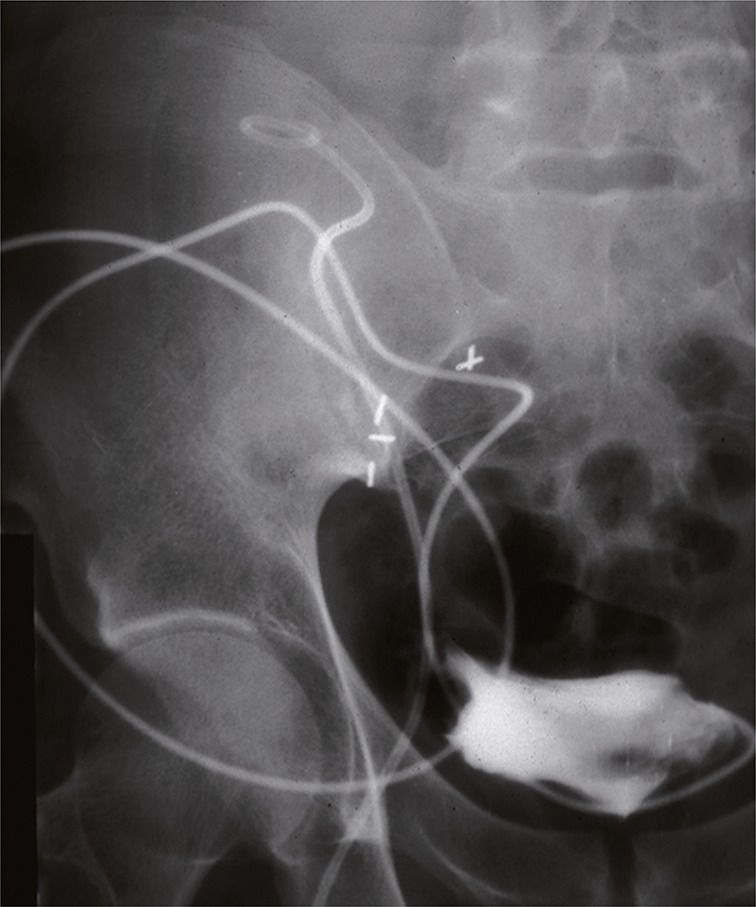
AP view: A double “J” stent from transplant kidney pelvis to bladder has been seated over the guide wire in the native ureter. The nephrostomy has been maintained as safety measure to ensure ready access.

A rigid ureteroscope was advanced under fluoroscopic guidance into the right native ureter, displacing the same toward the dilated superior hydrocalyx, which was then accessed by an 18 gauge needle advanced via the ureteroscope (Figure-3). A stiff Amplatz guide wire was then introduced into the kidney pelvis, over which a double “J” stent was placed, maintaining drainage from the kidney into the bladder (7) (Figure-4). The stent was maintained in position for 12 weeks. A solid fistula (uretero-calycostomy fistula) between calyx and native ureter resulted. Urine output of the transplant kidney stabilized; creatinine dropped to 2.6 in 4 weeks and remained stable at a level of 1.4 – 1.8 over the next 7 year follow-up, as did BUN at levels of 18 - 24.

Percutaneous radiologic antegrade uretero-neocalycostomy is recommended as a minimally invasive intervention that can manage the complex problem of ureter necrosis in otherwise well -functioning transplant kidneys.
